# Epidemiology and biology of physical activity and cancer recurrence

**DOI:** 10.1007/s00109-017-1558-9

**Published:** 2017-06-15

**Authors:** Christine M. Friedenreich, Eileen Shaw, Heather K. Neilson, Darren R. Brenner

**Affiliations:** 1 Department of Cancer Epidemiology and Prevention Research, CancerControl Alberta, Alberta Health Services, 2210 2nd St SW, Calgary, AB T2S 3C3 Canada; 2 Department of Oncology, Cumming School of Medicine, University of Calgary, Calgary, AB Canada; 3 Department of Community Health Sciences, Cumming School of Medicine, University of Calgary, Calgary, AB Canada

**Keywords:** Physical activity, Exercise, Recurrence, Biomechanisms, Cancer

## Abstract

Physical activity is emerging from epidemiologic research as a lifestyle factor that may improve survival from colorectal, breast, and prostate cancers. However, there is considerably less evidence relating physical activity to cancer recurrence and the biologic mechanisms underlying this association remain unclear. Cancer patients are surviving longer than ever before, and fear of cancer recurrence is an important concern. Herein, we provide an overview of the current epidemiologic evidence relating physical activity to cancer recurrence. We review the biologic mechanisms most commonly researched in the context of physical activity and cancer outcomes, and, using the example of colorectal cancer, we explore hypothesized mechanisms through which physical activity might intervene in the colorectal recurrence pathway. Our review highlights the importance of considering pre-diagnosis and post-diagnosis activity, as well as cancer stage and timing of recurrence, in epidemiologic studies. In addition, more epidemiologic research is needed with cancer recurrence as a consistently defined outcome studied separately from survival. Future mechanistic research using randomized controlled trials, specifically those demonstrating the exercise responsiveness of hypothesized mechanisms in early stages of carcinogenesis, are needed to inform recommendations about when to exercise and to anticipate additive or synergistic effects with other preventive behaviors or treatments.

## Introduction

Over the past two decades, it has become clear that physical activity is associated with reduced cancer incidence. A recent pooled analysis of epidemiologic studies showed that high levels of leisure-time physical activity are associated with statistically significantly lower risks of 10 different cancers, even after adjusting for body mass index (BMI) [1]. Achieving physical activity recommendations of the World Health Organization has been associated with a 7% decrease in overall cancer risk, with the strongest associations observed for colorectal cancer and female breast cancer [2]. Considerably fewer epidemiologic studies have investigated the role of physical activity in relation to cancer outcomes, and the majority examined mortality (overall and/or cancer-specific) as an end point, without analyzing recurrence [3]. Of 33 reviews we identified in the past decade that investigated physical activity and cancer survivorship, only eight reviewed recurrences as a distinct endpoint [4–11].

Cancer patients are surviving longer than ever before, and fear of cancer recurrence is an important concern among cancer survivors [12]. In the USA, an estimated 40% of patients treated for local and locally advanced colorectal cancer experience cancer recurrence [13] and, for breast cancer, recurrences affect 11–20% of patients depending on tumor characteristics, stage of cancer, and treatment [14]. In this review, we provide an overview of the understudied association between physical activity and cancer recurrence. We review and discuss hypothesized pathways and mechanisms that might explain epidemiologic findings, using the example of colorectal cancer. We also highlight gaps in knowledge and future directions for this area of research.

## Epidemiologic evidence

While there is strong and consistent observational evidence that increased physical activity is associated with increased overall survival in cancer survivors (mainly breast and colorectal [15]), as well as an inverse dose-response relationship with cancer-specific mortality [3], the evidence for physical activity in cancer recurrence is more limited (Table 1). Recurrence studies have been observational, mostly restricted to breast, colorectal, and prostate cancer survivor populations, and have found mixed results. There are several issues surrounding research on cancer recurrence that need to be recognized when examining the epidemiologic evidence. Cancer recurrence is difficult to study because most cancer registries do not routinely collect recurrence data [30]. Information on recurrence is generally only collected in clinical trials and is acquired through laborious chart reviews. In contrast, studies on cancer-specific and overall mortality are easier to conduct since these outcomes are readily accessible through tumor and vital registries and death certificates. Furthermore, varying definitions for cancer recurrence have been used which hinders comparisons between studies and may introduce error in the outcome assessments. For example, some definitions of recurrence outcomes include cancer-specific deaths or progressions (Table 1). Slow-developing recurrences are also difficult to assess because of the long follow-up required; observational studies might terminate active follow-up before a cancer recurrence is detected. In addition, studies may be underpowered to assess recurrence associations if the primary outcome (on which the sample size was based) is survival. Moreover, associations between recurrences and physical activity may be confounded by cancer symptoms or treatment, disease progression, and BMI.

**Table 1:** Summary of published epidemiologic studies relating physical activity to cancer recurrence by cancer site

Cancer site	First author (year), country	Study design	Cancer stage	Physical activity measure	Outcome	Results	
						PA category (MET-h/week)	HR (95% CI)
Breast	Jones (2016), USA [16]	Pooled analysis of two prospective cohorts (LACE and Pathways)	I–IIIa	Post-diagnosis recreational PA	Breast cancer recurrence assessed by self-report and Kaiser Permanente Northern California electronic medical record review	<2 2–10 >10–25 >25	1.00 1.00 (0.81–1.24) 0.92 (0.74–1.15) 1.01 (0.80–1.27)
	de Glas (2014), Netherlands [17]	Prospective cohort (TEAM-L)	0-–IV	Pre- and post-diagnosis recreational PA	Relapse-free period (time until disease recurrence or breast cancer death)	Pre-diagnosis: 0–22 22.4–41.5 41.6–70.8 >70.8 Post-diagnosis: 0–21.0 21.1–40.0 40.1–65.5 65.6–258	1.00 1.07 (0.55–2.11) 0.67 (0.33–1.38) 1.04 (0.53–2.02) 1.00 0.54 (0.23–1.29) 0.97 (0.44–2.13) 0.90 (0.39–2.10)
	Schmidt (2013), Germany [18]	Prospective cohort (MARIE)	I–IIIa	Pre-diagnosis recreational PA from age 50	Cancer recurrence (ipsilateral/contralateral/local/regional invasive recurrence or distant recurrence emerging after primary diagnosis)	None >0–<12 2–<24 24–<42 ≥42	1.00 0.96 (0.70–1.32) 0.93 (0.66–1.32) 0.97 (0.61–1.25) 0.65 (0.44–0.97)
	Friedenreich (2009), Canada [19]	Prospective cohort	0–IIIc	Pre-diagnosis lifetime total PA	Cancer recurrence, progression, or new primary cancer	<95 >120–≤151 >151	1.00 1.00 (0.73–1.37) 1.22 (0.89–1.68)
	Bao (2015), China [20]	Prospective cohort of triple-negative breast cancers	I-III	Post-diagnosis total PA (at 60 months post diagnosis)	Recurrence-/disease-specific mortality	None <7.6 ≥7.6	1.00 0.64 (0.39–1.07) 0.54 (0.35–0.84)
	Bertram (2011), USA [21]	Prospective cohort (WHEL)	I–III	Post-diagnosis total PA	Invasive breast cancer recurrence (local/regional or distal) or new primary breast cancer	0–2.5 2.5–7.5 7.5–14.9 14.9–24.7 24.7–107	1.00 0.91 (0.64–1.28) 0.85 (0.59–1.22) 0.97 (0.68–1.39) 0.74 (0.50–1.10)
	Chen (2011), China [22]	Prospective cohort	I-III	Post-diagnosis total PA (at 36 months post diagnosis)	Recurrence-/disease-specific mortality	None <8.3 ≥8.3	1.00 0.60 (0.46–0.78) 0.59 (0.45–0.76)
	Sternfeld (2009), USA [23]	Prospective cohort (LACE)	I–IIIa	Post-diagnosis total PA	Breast cancer recurrence (local, regional, or distant recurrence), metastasis, or death from breast cancer if no recurrence previously reported	<29 29–<44 44–<62 ≥62	1.00 0.76 (0.51–1.13) 0.87 (0.59–1.29) 0.91 (0.61–1.36)
	Holmes (2005), USA [24]	Prospective cohort (NHS)	I–III	Post-diagnosis leisure-time physical activity (after 2 years)	Breast cancer recurrence (second cancer diagnosis of lung, liver, bone, or brain) and breast cancer-specific deaths	<3 3–8.9 9–14.9 15–23.9 ≥24	1.00 0.83 (0.64–1.08) 0.57 (0.38–0.85) 0.66 (0.47–0.93) 0.74 (0.53–1.04)
	Courneya (2014), Canada [25]	Randomized controlled trial (START)	I–IIIA	Supervised aerobic or resistance exercise during chemotherapy	Recurrence-free interval (time to invasive ipsilateral breast tumor recurrence; local, regional, or distant recurrence; death from breast cancer)	Control Exercise groups	1.00 0.61 (0.31–1.21)
Colorectal	Walter (2017), Germany [26]	Prospective cohort (DACHS study)	I–IV	Lifetime and latest (past decade) pre-diagnostic leisure-time PA	Recurrence-free survival (self-reported or from last treating physician prior to death)	Lifetime: 0–25.4 >25.4–43.5 >43.5–65.4 >65.4 Latest: 0–13.2 >13.2–29.2 >29.2–56.2 >56.2	1.00 1.00 (0.82–1.23) 0.98 (0.80–1.19) 1.10 (0.90–1.33) 1.00 1.01 (0.83–1.23) 0.83 (0.68–1.02) 0.99 (0.81–1.22)
	Meyerhardt, 2006 [27]	Prospective cohort (CALGB 89803)	III	Post-diagnosis total PA	Recurrence-free survival (time to tumor recurrence or occurrence of a new primary colon tumor)	<3 3–8.9 9–17.9 18–26.9 ≥27	1.00 0.86 (0.57–1.30) 0.89 (0.55–1.42) 0.51 (0.26–1.01) 0.60 (0.36–1.01)
Prostate	Friedenreich (2016), Canada [28]	Prospective cohort (Alberta Prostate Cancer Cohort Study)	II–IV	Pre-diagnosis lifetime total PA and post-diagnosis total PA	Progression or recurrence (further disease, identified through PSA changes and secondary treatments following a significant disease-free period)	Pre-diagnosis: ≤98 >98–≤145 >145–≤199 >199 Post-diagnosis: ≤43 >43–≤75 >75–≤121 >121	1.00 0.80 (0.56–1.15) 0.84 (0.59–1.21) 0.94 (0.65–1.34) 1.00 1.10 (0.75–1.61) 1.31 (0.89–1.92) 1.23 (0.80–1.89)
	Richman (2011), USA [29]	Prospective cohort (CaPSURE substudy)	I–II	Post-diagnosis PA	Progression (biochemical recurrence, secondary treatment, or prostate cancer death)	Non-vigorous: 0–0.9 h/week 1.0–2.9 3.0–4.9 5.0–9.9 ≥10 Vigorous: 0 h/week 0.1–1.24 1.25–2.9 ≥3.0	1.00 0.80 (0.45–1.40) 0.90 (0.44–1.83) 0.71 (0.38–1.32 0.96 (0.52–1.76) 1.00 0.88 (0.55–1.41) 0.71 (0.40–1.28) 0.69 (0.35–1.36)

*PA* physical activity, *LACE* Life After Cancer Epidemiology, *TEAM-L* Tamoxifen Exemestane Adjuvant Multicenter Lifestyle, *WHEL* Women’s Healthy Eating and Living, *NHS* Nurses’ Health Study, *START* Supervised Trial of Aerobic versus Resistance Training, *DACHS* German: Darmkrebs: Chancen der Verhütung durch Screening, English: Colorectal Cancer: chances for prevention through screening, *CALGB* Cancer and Leukemia Group B, *PSA* prostate-specific antigen, *CaPSURE* Cancer of the Prostate Strategic Urologic Research Endeavor

To our knowledge, seven observational studies have investigated the effect of pre- or post-diagnosis physical activity and breast cancer events, defined as recurrence, progression, new primary breast cancers, or breast cancer-specific deaths [18–24]. A meta-analysis by Lahart et al. (2015) [10] provided pooled estimates for five of these studies that were protective (HR = 0.72, 95% CI 0.56–0.91 for pre-diagnosis physical activity [18, 19] and HR = 0.79, 95% CI 0.63–0.98 for post-diagnosis physical activity [21, 23, 24]), although most individual studies had non-statistically significant protective effects for cancer recurrence. Similarly, two observational studies of colorectal cancer found that increased pre- or post-diagnosis physical activity was not statistically significantly associated with recurrence-free survival, although results suggested a protective trend [26, 27]. For prostate cancer, two studies were conducted and no associations were observed between pre- and post-diagnosis physical activity with increased recurrence or progression of prostate cancer [28, 29]. There are currently several additional cohort studies in progress; two focus on colorectal cancer recurrence [31, 32] and two on breast cancer recurrence [33, 34]. These studies will collect repeated measures of physical activity over the course of follow-up and examine overall survival, disease-free survival, and/or recurrence as primary end points.

In addition to observational evidence, randomized controlled trials (RCTs) of exercise with recurrence outcomes are necessary for ruling out reverse causality, residual confounding, and evaluating the predictive nature of proposed biomarkers. There is emerging RCT evidence on the effect of post-diagnosis physical activity on cancer recurrence. To date, only the Supervised Trial of Aerobic versus Resistance Training (START) in breast cancer patients has published results, suggesting a protective exercise effect in patients diagnosed as human epidermal growth factor receptor 2 (HER2)-positive and patients who completed >85% of the average relative dose intensity of their originally planned chemotherapy regimen. However, the recurrence analysis from START was secondary and exploratory in nature and subgroups were based on small sample sizes [25]. Another trial in colon cancer patients that is ongoing is the Colon Health and Life-Long Exercise Change (CHALLENGE) Trial, designed specifically to evaluate the effect of post-diagnosis exercise on disease-free survival as a primary outcome [35]. There are currently a number of additional RCTs in cancer survivors investigating the effect of lifestyle (including physical activity) interventions on recurrence- or disease-free survival. One RCT in breast cancer patients involves an exercise plus diet for weight loss intervention [36], and three additional RCTs are ongoing that will test the relative impact of lifestyle interventions (generally diet, exercise, and vitamin D) [37–40] on breast cancer recurrence. In addition, there are several other registered trials currently ongoing that involve physical activity and cancer recurrence in breast (clincialtrials.gov identifiers: NCT02035631, NCT02786875, NCT03091842, NCT02750826, NCT02161900, NCT02240836), prostate (NCT02252484), ovarian (NCT02529150), endometrial (NCT03095664), and other cancer survivors (NCT02473003, NCT01693172). Results of these RCTs will provide further evidence of a causal association between physical activity (combined with other lifestyle changes) and cancer recurrence.

## Mechanisms

**Fig. 1:**
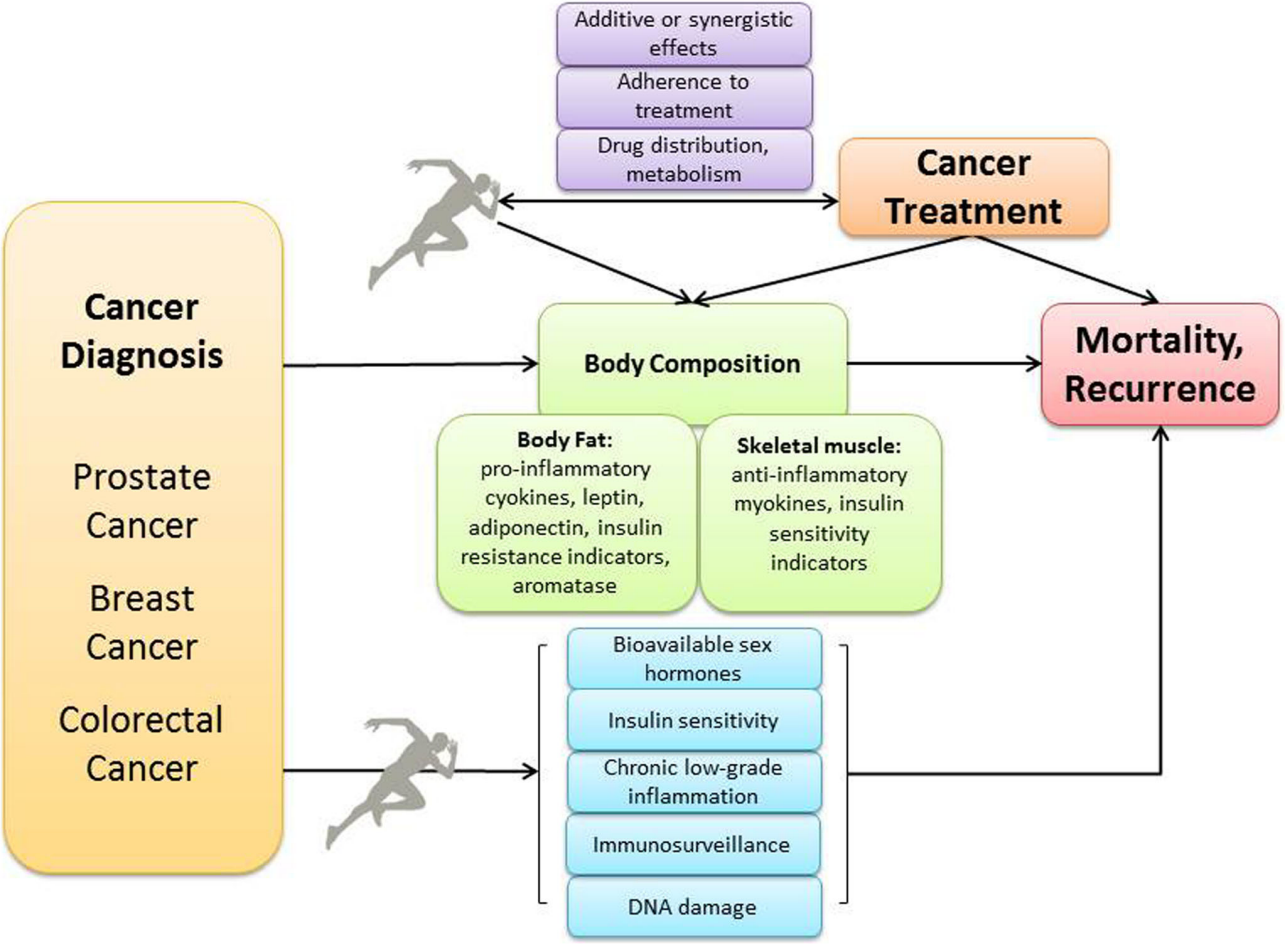
Commonly proposed mechanisms relating physical activity to cancer recurrence and/or survival. Potential additive or synergistic effects between physical activity and cancer treatment are possible

The mechanisms whereby physical activity could lower recurrence risk in breast, colorectal, and prostate cancer patients are not well understood, although evidence has grown over the past decade. The interrelated mechanisms most often studied in relation to physical activity and cancer prognosis (Fig. 1) include changes in whole-body and visceral fatness, metabolic dysregulation (e.g., insulin, glucose, insulin-like growth factors (IGF)), adipokines (e.g., leptin, adiponectin), and sex hormones (e.g., estrogen, testosterone); chronic, low-grade inflammation; oxidative stress causing DNA damage and gene mutations (e.g., tumor suppressor genes); and impaired immune surveillance/function [11, 41–43].

Whether physical activity influences these mechanisms independently or through reductions in adipose tissue volume and endocrine activity is difficult to discern for obesity-related cancers (e.g., colorectal, postmenopausal breast, prostate) since some of the same mechanisms are proposed, although some effects are becoming better understood. In postmenopausal women, for example, body fat is the primary source of endogenous estrogens, which fuel cancer progression in estrogen receptor-positive (ER+) breast cancer. There is now RCT evidence that, while exercise decreases estradiol levels, much greater decreases occur with weight loss [44] and exercise effects are at least partly mediated by fat loss [45]. Similarly, prolonged sedentary behavior (sitting or lying down) is a hypothesized cancer survival risk factor that may act independently of physical inactivity and body fat [46]. Yet, only in the past decade have epidemiologic studies begun to measure sedentary behavior as a distinct exposure.

Exercise intervention trials are increasingly being conducted to demonstrate exercise modes of action in cancer patients. Structured exercise trials (reviewed in [47, 48]), often in breast cancer patients, have shown mixed effects from exercise on circulating biomarkers such as metabolic factors (IGF-1 or its binding protein IGFBP-3, insulin, glucose, C-peptide, leptin), immune and inflammatory factors (natural killer cell cytotoxicity, pro- and anti-inflammatory cytokines), and measures of oxidative stress (8-oxo-dG, F2-isoprostane). More consistent exercise effects were found for circulating C-reactive protein levels (consistently decreased) and in prostate cancer patients, for testosterone- and prostate-specific antigen levels (consistently no change).

A common limitation of these trials is the uncertainty that circulating blood biomarkers reflect downstream biological activity, whereas epigenetic and gene expression studies do not have this limitation. For instance, in colorectal cancer patients, exercise was associated with the CpG island methylator phenotype (CIMP) and mutations in TP53 and KRAS2 mutations in colon tumor tissue [49] and CIMP-positive tumors are associated with reduced survival [50]. Future exercise trials could potentially measure changes in microRNA, global DNA methylation, and gene-specific methylation, since these outcomes were associated with exercise in previous studies [51, 52]; e.g., higher levels of physical activity have been associated with less frequent CACNA2D3 methylation in gastric adenocarcinoma patients in an observational study [53].

Exercise during first-line and adjuvant chemotherapy might improve treatment efficacy (Fig. 1). Shared mechanisms between exercise and cancer treatment, such as weight loss and decreased sex hormones from exercise combined with the use of aromatase inhibitors, or improved immune function combined with immunotherapy, may generate additive or synergistic improvements. Furthermore, exercise during chemotherapy has been shown to improve adherence to treatment [25, 54].

### The importance of timing and stage

A key consideration related to physical activity and cancer recurrence is timing. There is clinical value in understanding whether pre- or post-diagnosis physical activity (or both) prevents cancer recurrence, and whether physical activity prevents early or late recurrences (or both). Early recurrences of a slow-growing cancer could be more strongly influenced by pre-diagnosis activity whereas late recurrences could be more strongly influenced by post-diagnosis activity. Moreover, if risk of recurrence increases with cancer stage at first diagnosis [55], then physical activity mechanistic research should also be stage-specific. This knowledge would help guide mechanistic studies to target early or late carcinogenesis. Ultimately, research to understand the sequence of risk accumulation—and physical activity’s role at each step in the sequence—will guide prevention messaging and intervention studies, including when to intervene, what benefit to expect, and for what outcome [56].

### Colorectal cancer recurrence

**Fig. 2:**
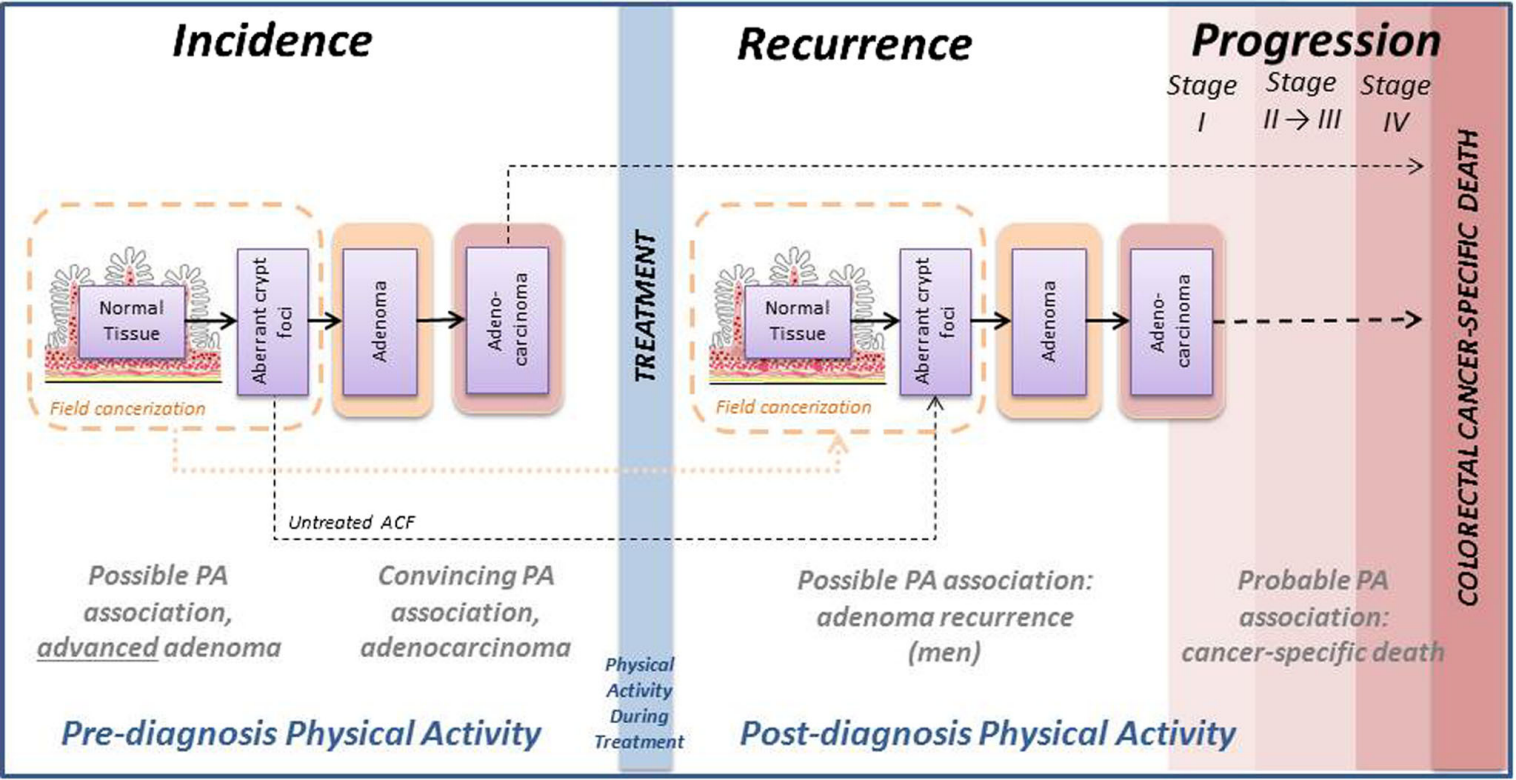
Colorectal cancer serves as a useful example for studying physical activity mechanisms because risk accumulation is observable. The overall strength and consistency of epidemiologic evidence relating physical activity to colorectal cancer outcomes is strongest for colorectal cancer incidence, somewhat weaker for colorectal cancer mortality (given fewer prospective studies; although results have been relatively consistent, generally showing benefit from both pre- and post-diagnosis physical activity), and weakest for adenoma (polyp) recurrence, due to limited epidemiologic research focused on recurrence outcomes and studies showing significant associations only in men. *Solid arrows* indicate known pathways. *Broken arrows* indicate hypothesized pathways. *ACF*, aberrant crypt foci; *PA*, physical activity

Colorectal cancer serves as a useful example for aligning physical activity timing with risk accumulation because in colorectal cancer the early stages are observable; from field cancerization [57, 58] to aberrant crypt foci, to adenomatous polyps, to adenocarcinoma (95% of cases), to cancer-specific death (Fig. 2). Colorectal cancer may develop over 10 years, resulting from the accumulation of multiple gene mutations—including *APC* and *KRAS* followed by *PIK3CA*, *SMAD4*, *TP53*, and others [59], with only a small proportion of aberrant crypt foci ever progressing to cancer [60]. Recurrences occur in ~40% of colorectal cases in the USA [13] and happen more quickly than for other cancer sites, with the majority (80–95%) appearing within 5 years of surgical resection [61]. Given that pre- and post-diagnosis physical activities are associated with better colorectal cancer survival outcomes [62], it is plausible that physical activity influences early- and late-stage mechanisms. Below, we review mechanisms previously proposed for physical activity and colorectal cancer recurrence by stage of carcinogenesis, namely, (1) field cancerization, (2) adenoma recurrence, and (3) adenocarcinoma recurrence.

#### Field cancerization of the colon

Field cancerization [63] refers to the concept of an area of otherwise normal epithelial tissue that is ‘primed’ to undergo transformation (e.g., by methylated DNA [64], altered gene expression [65], etc.), which can either be locally focused or diffuse in an organ such as the colon [58]. This phenomenon may explain colorectal cancer recurrence, with new cancers potentially arising either adjacent to the excised primary tumor or more diffusely where a broader field is observed. There is some evidence from animal studies that exercise can favorably alter DNA methylation patterns. For example, repression of the gene BHMT2, involved in aberrant methylation, was observed in the colons of exercising rats [66]. However, there is inherent complexity with these types of investigations in humans, and therefore, the human evidence to date has been limited with mixed results [46]. In several observational studies, increased physical activity was associated with higher levels of global methylation in peripheral blood [67, 68] and exercise was shown to impact levels of global DNA methylation in adipose tissue in an intervention study [69]. However, no intervention trials have examined if exercise induces changes in DNA methylation in colon tissue. Furthermore, no studies have investigated if exercise can reverse the cancerization of a particular field with repeated measures in colon tissues.

#### Recurrence of adenomas (polyps)

A number of epidemiologic studies have examined the relation between physical activity [70–72] or sedentary behavior [73–75] and adenoma risk. These studies generally found a benefit from being more active, particularly for adenomas that were large/more advanced. Fewer studies focused on adenoma recurrence [75–78] and, of those, some suggested lower recurrence risk associated with more activity [77] or less sedentary behavior [75] in men.

Physical activity might prevent adenoma recurrence by maintaining energy balance since BMI, waist circumference, and possibly weight change have been associated with higher recurrence risk [79–82]. Correlative studies suggest that adenoma recurrence could be mediated partly by homocysteine levels [78], age, blood glucose [83], metabolic syndrome, waist circumference, or waist-to-hip ratio, particularly in men [84]. An inflammatory mechanism (e.g., via COX-2 or STAT3) is plausible since aspirin use [85] and various cytokines [86] are associated with adenoma risk. Adipokines such as leptin and adiponectin, derived from white adipose tissue, are also hypothesized to play direct or indirect opposing roles (leptin unfavorable, adiponectin favorable) mediating cancer cell proliferation, invasion, and survival [87]. However, one epidemiologic study of adenoma recurrence found risk to be *lower* among individuals with higher leptin levels and there was no association with adiponectin [78].

Few epidemiologic studies have explored insulin resistance biomarkers in relation to adenoma recurrence despite insulin’s known mitogenic and anti-apoptotic properties in the colon [88] and exercise responsiveness in other contexts. One epidemiologic study reported an increased risk of adenoma recurrence in adults with higher levels of circulating glucose and insulin [89], although C-peptide, a by-product of insulin secretion, was not associated with recurrence in another study [78].

Other epidemiologic studies investigated IGF-1 and its primary binding protein IGFBP-3, which controls IGF-1 bioavailability, in relation to adenoma recurrence since IGF-1 promotes cell proliferation and inhibits apoptosis [90]. In some prospective studies, inverse associations between baseline IGF-1 levels and future adenoma recurrence were reported, perhaps because of better health in the higher-level IGF-1 subgroups [91, 92]. However, the impact of exercise on IGF is most likely to be indirect by altering, for example, IGF-1 physiology or through energy balance [93].

#### Recurrence of adenocarcinoma (cancer)

The mechanisms most commonly proposed for colorectal cancer recurrence, besides tumor stage, location, and cancer treatment, include *KRAS* and mismatch repair gene mutations, microsatellite instability status [94], and MCMT promoter methylation [95]. Furthermore, many of the same mechanisms proposed for adenoma recurrence are proposed for cancer recurrence; these mechanisms and others are discussed below.

Diabetic patients and those with increased number of metabolic syndrome conditions are at increased risk of colorectal cancer recurrence (or reduced recurrence/disease-free survival) [96, 97], suggesting a role for metabolic dysregulation. There is inconsistent evidence that increased insulin and IGF-1 are associated with worse prognosis in colorectal cancer patients [98–100]. Higher IGFBP-3 levels may be associated with a reduced risk of colorectal cancer-specific death [100]. However, these associations have not been well-studied for recurrence. Only one study by Lee et al. [48] investigated the effect of a physical activity intervention on the IGF-1 axis in colorectal cancer patients. Results indicated that increased physical activity significantly decreased insulin levels and homeostasis model assessment of insulin resistance and increased IGF-1 and IGFBP-3 levels. These results support the IGF-1 axis as a potential mechanism underlying physical activity benefits, with the exception of the increase in IGF-1 levels, which the authors explained by a correlation between IGF-1 and lean mass at baseline.

Tumor-promoting inflammation is an enabling characteristic of cancer [101] that has been implicated in colorectal cancer recurrence [102], yet it is unclear if exercise is protective. In rats, exercise has attenuated chemically induced COX-2 expression and cell proliferation in the colon [103], modeling early stage prevention. Myokines might be involved, which are cytokines and other peptides released from muscle cells that promote anti-inflammatory effects and insulin sensitivity; interleukin-6 is the most commonly studied [42, 104]. Secreted protein acidic and rich in cysteine (SPARC) is a myokine recently studied in relation to early colorectal carcinogenesis that prevented aberrant crypt formation in mice [105]. However, a small trial in humans did not find SPARC levels to be exercise-responsive [106]. A commonly studied pro-inflammatory cancer biomarker is TNF-α. Higher TNF-α expression in colon tumor tissue has been associated with positive lymph node stage and colon cancer recurrence [107], and there is some evidence that circulating TNF-α levels can be lowered with exercise in animal models [108] and in colon cancer patients [48] although this effect is not found consistently in cancer patients [47].

Chronic exercise might decrease recurrence risk by lowering systemic oxidative stress, resulting from immune cell overproduction of reactive oxygen species (ROS). The genetic and epigenetic changes induced by ROS may contribute to the initiation and progression of colorectal cancer [109]. Chronic exercise could reduce oxidative stress by inducing an adaptive response since exercise itself induces ROS production in skeletal muscle [110]. Alternatively, exercise may decrease ROS exposure by lowering hydrophobic bile acid concentrations [111] perhaps via decreased serum cholesterol [112]. Recently, a large RCT in overweight/obese postmenopausal women showed a significant decrease in circulating F_2_-isoprostane levels after 12 months of exercise, but no change in fluorescent oxidation products or oxidized low-density lipoprotein levels [113].

Multiple epigenetic alterations have been implicated in the development and prognosis [114] of colorectal adenocarcinoma. These include but are not limited to the CIMP phenotype [50] (often identified through the methylation of RUNX3, SOCS1, NEUROG1, CACNA1G, and IGF) [115], global hypomethylation and hypermethylation of tumor suppressor genes (CDKN2A and ESR, APC, KRAS, MGMT) [116], and alterations in microRNA (miRNA) expression [117]. While the impact of these epigenetic alterations on prognosis has been relatively well characterized, given the challenges in adequately measuring exercise in cancer patients in observational studies, the impact of exercise has been mixed [118]. One study examined gene expression in the colons of exercising rats and discovered reduced transcript levels for VEGF (vascularization), ANG-2 (vascularization), and iPL-A2 (signal transduction) [66]. While epigenetic markers such as miRNA panels provide interesting etiologic candidates in observational studies, several limitations to their use should be noted. For the example of miRNA, major limitations include variability in miRNA isolation and extraction [119], as well as the cross-platform variation in results from miRNA quantification [120]. Furthermore, within-platform differences have been observed across commercial offerings, which further limits their broad applicability [121]. As the research advances, additional attention should be given to standardization of sample collection and storage as well as of methods for quantification and analyses (normalization).

### Colorectal cancer recurrence: when could exercise be beneficial?

Our review of physical activity and colorectal cancer mechanisms shows that, to date, the strongest evidence relating physical activity to colorectal cancer recurrence in humans relates to “near-diagnosis” processes (i.e., not initiation, not late-stage), namely, weight control and insulin-related pathways. Inflammatory pathways could mediate adenoma (polyp) recurrence in some subgroups (e.g., men), although clarity is needed to understand whether physical activity, sedentary behavior, or body fatness is driving this mediation.

Perhaps the greatest immediate opportunity for mechanistic research relates to the role of exercise in early, initiating events in colorectal cancer (e.g., preventing field cancerization and aberrant crypt foci). Currently, there is very little evidence in humans that exercise modulates these events. Late-stage carcinogenic pathways are also not well understood, in part because epidemiologic survival studies of physical activity often exclude stage IV cases (Table 1) to avoid possible reverse causation, and trials to understand exercise modes of action have been infrequently conducted in this group. Although tumor-promoting inflammation and avoiding immune destruction are enabling characteristics of cancer [101], there is still very little direct evidence that exercise alters these pathways in the colon. Comparatively more evidence suggests insulin-glucose regulation may be involved in adenocarcinoma recurrence.

## Future directions

Presently, there is limited epidemiologic evidence relating physical activity to cancer recurrence. Rather, benefit is inferred from cancer survival studies which often focus on post-diagnosis physical activity in breast, colorectal, and prostate cancer survivors. There may be benefit from considering pre-diagnosis activity as well as cancer stage at diagnosis and timing of recurrence (early/late) in future epidemiologic studies, to guide mechanistic research. In addition, standardized, cancer-specific definitions of recurrence are needed to build a more consistent body of epidemiologic evidence regarding recurrence. The possibility that physical activity-recurrence associations are partly explained by body fatness and sedentary behavior, or perhaps diet quality [11], must also be carefully considered when interpreting observational studies. Ideally, future observational cohort studies of physical activity and cancer recurrence should be designed to include objective measurements of physical activity, sedentary behavior, body composition, other health-related factors, and biologic mechanisms within the same study population as is being done in the ongoing Alberta Moving Beyond Breast Cancer cohort study [122].

There are some indications about how physical activity might influence cancer recurrence mechanisms, although for some mechanisms (e.g., IGF, oxidative stress, epigenetic mechanisms) there is uncertainty regarding whether or not they are modifiable with exercise while for others (e.g., circulating biomarkers), it is unclear if they are strongly predictive of recurrence. Research from RCTs that demonstrate both exercise responsiveness and clinical relevance is needed. Furthermore, studies that examine the impact of exercise on the reversibility of field cancerization or early carcinogenic events (e.g., in colorectal cancer), with repeated longitudinal sampling from tissues of interest, are needed.

Although our recurrence model focused on colorectal cancer, the same thought process of considering mechanisms separately for precancerous outcomes is useful for studying the role of lifestyle factors in the recurrence of other slow-growing cancers. This level of mechanistic insight is crucial for informing recommendations about when to exercise and for anticipating additive or synergistic effects with other preventive behaviors or treatments.

## References

[1] Moore SC, Lee IM, Weiderpass E, Campbell PT, Sampson JN, Kitahara CM, Keadle SK, Arem H, Berrington de Gonzalez A, Hartge P (2016). Association of leisure-time physical activity with risk of 26 types of cancer in 1.44 million adults. JAMA Intern Med.

[2] Liu L, Shi Y, Li T, Qin Q, Yin J, Pang S, Nie S, Wei S (2016). Leisure time physical activity and cancer risk: evaluation of the WHO's recommendation based on 126 high-quality epidemiological studies. Br J Sports Med.

[3] Li T, Wei S, Shi Y, Pang S, Qin Q, Yin J, Deng Y, Chen Q, Wei S, Nie S (2016). The dose-response effect of physical activity on cancer mortality: findings from 71 prospective cohort studies. Br J Sports Med.

[4] Harris SR (2009). Physical activity and breast cancer mortality. Eur J Oncol Nurs.

[5] Ibrahim EM, Al-Homaidh A (2011). Physical activity and survival after breast cancer diagnosis: meta-analysis of published studies. Med Oncol.

[6] Ligibel J (2012). Lifestyle factors in cancer survivorship. J Clin Oncol.

[7] Loprinzi PD, Cardinal BJ, Winters-Stone K, Smit E, Loprinzi CL (2012). Physical activity and the risk of breast cancer recurrence: a literature review. Oncol Nurs Forum.

[8] Ellsworth RE, Valente AL, Shriver CD, Bittman B, Ellsworth DL (2012). Impact of lifestyle factors on prognosis among breast cancer survivors in the USA. Expert Rev Pharmacoecon Outcomes Res.

[9] Chlebowski RT (2013). Nutrition and physical activity influence on breast cancer incidence and outcome. Breast.

[10] Lahart IM, Metsios GS, Nevill AM, Carmichael AR (2015). Physical activity, risk of death and recurrence in breast cancer survivors: a systematic review and meta-analysis of epidemiological studies. Acta Oncol.

[11] Dieli-Conwright CM, Lee K, Kiwata JL (2016). Reducing the risk of breast cancer recurrence: an evaluation of the effects and mechanisms of diet and exercise. Curr Breast Cancer Rep.

[12] Simard S, Thewes B, Humphris G, Dixon M, Hayden C, Mireskandari S, Ozakinci G (2013). Fear of cancer recurrence in adult cancer survivors: a systematic review of quantitative studies. J Cancer Surviv.

[13] Siegel R, DeSantis C, Virgo K, Stein K, Mariotto A, Smith T, Cooper D, Gansler T, Lerro C, Fedewa S (2012). Cancer treatment and survivorship statistics, 2012. CA Cancer J Clin.

[14] Brewster AM, Hortobagyi GN, Broglio KR, Kau SW, Santa-Maria CA, Arun B, Buzdar AU, Booser DJ, Valero V, Bondy M (2008). Residual risk of breast cancer recurrence 5 years after adjuvant therapy. J Natl Cancer Inst.

[15] Schmid D, Leitzmann MF (2014). Association between physical activity and mortality among breast cancer and colorectal cancer survivors: a systematic review and meta-analysis. Ann Oncol.

[16] Jones LW, Kwan ML, Weltzien E, Chandarlapaty S, Sternfeld B, Sweeney C, Bernard PS, Castillo A, Habel LA, Kroenke CH (2016). Exercise and prognosis on the basis of clinicopathologic and molecular features in early-stage breast cancer: the LACE and pathways studies. Cancer Res.

[17] de Glas NA, Fontein DB, Bastiaannet E, Pijpe A, De Craen AJ, Liefers GJ, Nortier HJ, de Haes HJ, van de Velde CJ, van Leeuwen FE (2014). Physical activity and survival of postmenopausal, hormone receptor-positive breast cancer patients: results of the tamoxifen Exemestane adjuvant multicenter lifestyle study. Cancer.

[18] Schmidt ME, Chang-Claude J, Vrieling A, Seibold P, Heinz J, Obi N, Flesch-Janys D, Steindorf K (2013). Association of pre-diagnosis physical activity with recurrence and mortality among women with breast cancer. Int J Cancer.

[19] Friedenreich CM, Gregory J, Kopciuk KA, Mackey JR, Courneya KS (2009). Prospective cohort study of lifetime physical activity and breast cancer survival. Int J Cancer.

[20] Bao PP, Zhao GM, Shu XO, Peng P, Cai H, Lu W, Zheng Y (2015). Modifiable lifestyle factors and triple-negative breast cancer survival: a population-based prospective study. Epidemiology.

[21] Bertram LA, Stefanick ML, Saquib N, Natarajan L, Patterson RE, Bardwell W, Flatt SW, Newman VA, Rock CL, Thomson CA (2011). Physical activity, additional breast cancer events, and mortality among early-stage breast cancer survivors: findings from the WHEL Study. Cancer Causes Control.

[22] Chen X, Lu W, Zheng W, Gu K, Matthews CE, Chen Z, Zheng Y, Shu XO (2011). Exercise after diagnosis of breast cancer in association with survival. Cancer Prev Res (Phila).

[23] Sternfeld B, Weltzien E, Quesenberry CP, Castillo AL, Kwan M, Slattery ML, Caan BJ (2009). Physical activity and risk of recurrence and mortality in breast cancer survivors: findings from the LACE study. Cancer Epidemiol Biomark Prev.

[24] Holmes MD, Chen WY, Feskanich D, Kroenke CH, Colditz GA (2005). Physical activity and survival after breast cancer diagnosis. JAMA.

[25] Courneya KS, Segal RJ, McKenzie DC, Dong H, Gelmon K, Friedenreich CM, Yasui Y, Reid RD, Crawford JJ, Mackey JR (2014). Effects of exercise during adjuvant chemotherapy on breast cancer outcomes. Med Sci Sports Exerc.

[26] Walter V, Jansen L, Knebel P, Chang-Claude J, Hoffmeister M, Brenner H (2017) Physical activity and survival of colorectal cancer patients: population-based study from Germany. Int J Cancer. doi:10.1002/ijc.30619 10.1002/ijc.30619 28120416

[27] Meyerhardt JA, Heseltine D, Niedzwiecki D, Hollis D, Saltz LB, Mayer RJ, Thomas J, Nelson H, Whittom R, Hantel A (2006). Impact of physical activity on cancer recurrence and survival in patients with stage III colon cancer: findings from CALGB 89803. J Clin Oncol.

[28] Friedenreich CM, Wang Q, Neilson HK, Kopciuk KA, McGregor SE, Courneya KS (2016). Physical activity and survival after prostate cancer. Eur Urol.

[29] Richman EL, Kenfield SA, Stampfer MJ, Paciorek A, Carroll PR, Chan JM (2011). Physical activity after diagnosis and risk of prostate cancer progression: data from the cancer of the prostate strategic urologic research endeavor. Cancer Res.

[30] Warren JL, Yabroff KR (2015) Challenges and opportunities in measuring cancer recurrence in the United States. J Natl Cancer Inst:107. doi:10.1093/jnci/djv134 10.1093/jnci/djv134 PMC4580558 25971299

[31] Winkels RM, Heine-Broring RC, van Zutphen M, van Harten-Gerritsen S, Kok DE, van Duijnhoven FJ, Kampman E (2014). The COLON study: Colorectal cancer: Longitudinal, Observational study on Nutritional and lifestyle factors that may influence colorectal tumour recurrence, survival and quality of life. BMC Cancer.

[32] Soares-Miranda L, Abreu S, Silva M, Peixoto A, Ramalho R, da Silva PC, Costa C, Teixeira JP, Goncalves C, Moreira P (2017). Cancer Survivor Study (CASUS) on colorectal patients: longitudinal study on physical activity, fitness, nutrition, and its influences on quality of life, disease recurrence, and survival. Rationale and design. Int J Color Dis.

[33] Taira N, Akiyama I, Ishihara S, Ishibe Y, Kawasaki K, Saito M, Shien T, Nomura T, Hara F, Mizoo T (2015). Impact of modifiable lifestyle factors on outcomes after breast cancer diagnosis: the Setouchi Breast Cancer Cohort Study. Jpn J Clin Oncol.

[34] Islam T, Bhoo-Pathy N, Su TT, Majid HA, Nahar AM, Ng CG, Dahlui M, Hussain S, Cantwell M, Murray L (2015). The Malaysian Breast Cancer Survivorship Cohort (MyBCC): a study protocol. BMJ Open.

[35] Courneya KS, Booth CM, Gill S, O'Brien P, Vardy J, Friedenreich CM, Au HJ, Brundage MD, Tu D, Dhillon H (2008). The Colon Health and Life-Long Exercise Change trial: a randomized trial of the National Cancer Institute of Canada Clinical Trials Group. Curr Oncol.

[36] Rock CL, Byers TE, Colditz GA, Demark-Wahnefried W, Ganz PA, Wolin KY, Elias A, Krontiras H, Liu J, Naughton M (2013). Reducing breast cancer recurrence with weight loss, a vanguard trial: the Exercise and Nutrition to Enhance Recovery and Good Health for You (ENERGY) Trial. Contemp Clin Trials.

[37] Rack B, Andergassen U, Neugebauer J, Salmen J, Hepp P, Sommer H, Lichtenegger W, Friese K, Beckmann MW, Hauner D (2010). The German SUCCESS C study—the first European lifestyle study on breast cancer. Breast Care (Basel).

[38] Arun B, Austin T, Babiera GV, Basen-Engquist K, Carmack CL, Chaoul A, Cohen L, Connelly L, Haddad R, Harrison C et al (2016) A comprehensive lifestyle randomized clinical trial: design and initial patient experience. Integr Cancer Ther. doi:10.1177/1534735416679516 10.1177/1534735416679516 PMC5558265 27903842

[39] Augustin LS, Libra M, Crispo A, Grimaldi M, De Laurentiis M, Rinaldo M, D'Aiuto M, Catalano F, Banna G, Ferrau F (2017). Low glycemic index diet, exercise and vitamin D to reduce breast cancer recurrence (DEDiCa): design of a clinical trial. BMC Cancer.

[40] Villarini A, Pasanisi P, Traina A, Mano MP, Bonanni B, Panico S, Scipioni C, Galasso R, Paduos A, Simeoni M (2012). Lifestyle and breast cancer recurrences: the DIANA-5 trial. Tumori.

[41] Koelwyn GJ, Wennerberg E, Demaria S, Jones LW (2015). Exercise in regulation of inflammation-immune axis function in cancer initiation and progression. Oncology (Williston Park).

[42] Mathur N, Pedersen BK (2008). Exercise as a mean to control low-grade systemic inflammation. Mediat Inflamm.

[43] Champ CE, Francis L, Klement RJ, Dickerman R, Smith RP (2016). Fortifying the treatment of prostate cancer with physical activity. Prostate Cancer.

[44] Campbell KL, Foster-Schubert KE, Alfano CM, Wang CC, Wang CY, Duggan CR, Mason C, Imayama I, Kong A, Xiao L (2012). Reduced-calorie dietary weight loss, exercise, and sex hormones in postmenopausal women: randomized controlled trial. J Clin Oncol.

[45] Friedenreich CM, Woolcott CG, McTiernan A, Ballard-Barbash R, Brant RF, Stanczyk FZ, Terry T, Boyd NF, Yaffe MJ, Irwin ML (2010). Alberta physical activity and breast cancer prevention trial: sex hormone changes in a year-long exercise intervention among postmenopausal women. J Clin Oncol.

[46] Hibler E (2015). Epigenetics and colorectal neoplasia: the evidence for physical activity and sedentary behavior. Curr Colorectal Cancer Rep.

[47] Betof AS, Dewhirst MW, Jones LW (2013). Effects and potential mechanisms of exercise training on cancer progression: a translational perspective. Brain Behav Immun.

[48] Lee DH, Kim JY, Lee MK, Lee C, Min JH, Jeong DH, Lee JW, Chu SH, Meyerhardt JA, Ligibel J (2013). Effects of a 12-week home-based exercise program on the level of physical activity, insulin, and cytokines in colorectal cancer survivors: a pilot study. Support Care Cancer.

[49] Slattery ML, Curtin K, Wolff RK, Herrick JS, Caan BJ, Samowitz W (2010). Diet, physical activity, and body size associations with rectal tumor mutations and epigenetic changes. Cancer Causes Control.

[50] Phipps AI, Limburg PJ, Baron JA, Burnett-Hartman AN, Weisenberger DJ, Laird PW, Sinicrope FA, Rosty C, Buchanan DD, Potter JD (2015). Association between molecular subtypes of colorectal cancer and patient survival. Gastroenterology.

[51] Tonevitsky AG, Maltseva DV, Abbasi A, Samatov TR, Sakharov DA, Shkurnikov MU, Lebedev AE, Galatenko VV, Grigoriev AI, Northoff H (2013). Dynamically regulated miRNA-mRNA networks revealed by exercise. BMC Physiol.

[52] Voisin S, Eynon N, Yan X, Bishop DJ (2015). Exercise training and DNA methylation in humans. Acta Physiol (Oxf).

[53] Yuasa Y, Nagasaki H, Akiyama Y, Hashimoto Y, Takizawa T, Kojima K, Kawano T, Sugihara K, Imai K, Nakachi K (2009). DNA methylation status is inversely correlated with green tea intake and physical activity in gastric cancer patients. Int J Cancer.

[54] Jones LW, Dewhirst MW (2014). Therapeutic properties of aerobic training after a cancer diagnosis: more than a one-trick pony?. J Natl Cancer Inst.

[55] O'Connell MJ, Campbell ME, Goldberg RM, Grothey A, Seitz JF, Benedetti JK, Andre T, Haller DG, Sargent DJ (2008). Survival following recurrence in stage II and III colon cancer: findings from the ACCENT data set. J Clin Oncol.

[56] Wei EK, Wolin KY, Colditz GA (2010). Time course of risk factors in cancer etiology and progression. J Clin Oncol.

[57] Shen L, Kondo Y, Rosner GL, Xiao L, Hernandez NS, Vilaythong J, Houlihan PS, Krouse RS, Prasad AR, Einspahr JG (2005). MGMT promoter methylation and field defect in sporadic colorectal cancer. JNCI: J Natl Cancer Inst.

[58] Luo Y, Yu M, Grady WM (2014). Field cancerization in the colon: a role for aberrant DNA methylation?. Gastroenterol Rep (Oxf).

[59] Vogelstein B, Papadopoulos N, Velculescu VE, Zhou S, Diaz LA, Kinzler KW (2013). Cancer genome landscapes. Science.

[60] Alrawi SJ, Schiff M, Carroll RE, Dayton M, Gibbs JF, Kulavlat M, Tan D, Berman K, Stoler DL, Anderson GR (2006). Aberrant crypt foci. Anticancer Res.

[61] Makhoul R, Alva S, Wilkins KB (2015). Surveillance and survivorship after treatment for colon cancer. Clin Colon Rectal Surg.

[62] Je Y, Jeon JY, Giovannucci EL, Meyerhardt JA (2013). Association between physical activity and mortality in colorectal cancer: a meta-analysis of prospective cohort studies. Int J Cancer.

[63] Slaughter DP, Southwick HW, Smejkal W (1953). Field cancerization in oral stratified squamous epithelium; clinical implications of multicentric origin. Cancer.

[64] Grady WM (2005). Epigenetic events in the colorectum and in colon cancer. Biochem Soc Trans.

[65] Jothy S, Flanders TY, Nowacki PM (1996). New developments in the molecular pathology of human colon cancer: relevance to pathogenesis and diagnosis. Adv Anat Pathol.

[66] Buehlmeyer K, Doering F, Daniel H, Kindermann B, Schulz T, Michna H (2008). Alteration of gene expression in rat colon mucosa after exercise. Ann Anat.

[67] Luttropp K, Nordfors L, Ekstrom TJ, Lind L (2013). Physical activity is associated with decreased global DNA methylation in Swedish older individuals. Scand J Clin Lab Invest.

[68] White AJ, Sandler DP, Bolick SC, Xu Z, Taylor JA, DeRoo LA (2013). Recreational and household physical activity at different time points and DNA global methylation. Eur J Cancer.

[69] Ronn T, Volkov P, Davegardh C, Dayeh T, Hall E, Olsson AH, Nilsson E, Tornberg A, Dekker Nitert M, Eriksson KF (2013). A six months exercise intervention influences the genome-wide DNA methylation pattern in human adipose tissue. PLoS Genet.

[70] Wolin KY, Yan Y, Colditz GA (2011). Physical activity and risk of colon adenoma: a meta-analysis. Br J Cancer.

[71] Song JH, Kim YS, Yang SY, Chung SJ, Park MJ, Lim SH, Yim JY, Kim JS, Jung HC (2013). Physical activity and other lifestyle factors in relation to the prevalence of colorectal adenoma: a colonoscopy-based study in asymptomatic Koreans. Cancer Causes Control.

[72] Sanchez NF, Stierman B, Saab S, Mahajan D, Yeung H, Francois F (2012). Physical activity reduces risk for colon polyps in a multiethnic colorectal cancer screening population. BMC Res Notes.

[73] Cao Y, Keum NN, Chan AT, Fuchs CS, Wu K, Giovannucci EL (2015). Television watching and risk of colorectal adenoma. Br J Cancer.

[74] Cao Y, Rosner BA, Ma J, Tamimi RM, Chan AT, Fuchs CS, Wu K, Giovannucci EL (2015). Assessing individual risk for high-risk colorectal adenoma at first-time screening colonoscopy. Int J Cancer.

[75] Molmenti CL, Hibler EA, Ashbeck EL, Thomson CA, Garcia DO, Roe D, Harris RB, Lance P, Cisneroz M, Martinez ME (2014). Sedentary behavior is associated with colorectal adenoma recurrence in men. Cancer Causes Control.

[76] Colbert LH, Lanza E, Ballard-Barbash R, Slattery ML, Tangrea JA, Caan B, Paskett ED, Iber F, Kikendall W, Lance P (2002). Adenomatous polyp recurrence and physical activity in the Polyp Prevention Trial (United States). Cancer Causes Control.

[77] Wallace K, Baron JA, Karagas MR, Cole BF, Byers T, Beach MA, Pearson LH, Burke CA, Silverman WB, Sandler RS (2005). The association of physical activity and body mass index with the risk of large bowel polyps. Cancer Epidemiol Biomark Prev.

[78] Bobe G, Murphy G, Rogers CJ, Hance KW, Albert PS, Laiyemo AO, Sansbury LB, Lanza E, Schatzkin A, Cross AJ (2010). Serum adiponectin, leptin, C-peptide, homocysteine, and colorectal adenoma recurrence in the Polyp Prevention Trial. Cancer Epidemiol Biomark Prev.

[79] Kitahara CM, Berndt SI, de Gonzalez AB, Coleman HG, Schoen RE, Hayes RB, Huang WY (2013). Prospective investigation of body mass index, colorectal adenoma, and colorectal cancer in the prostate, lung, colorectal, and ovarian cancer screening trial. J Clin Oncol.

[80] Jacobs ET, Martinez ME, Alberts DS, Jiang R, Lance P, Lowe KA, Thompson PA (2007). Association between body size and colorectal adenoma recurrence. Clin Gastroenterol Hepatol.

[81] Kim TJ, Kim JE, Choi YH, Hong SN, Kim YH, Chang DK, Rhee PL, Kim MJ, Jung SH, Son HJ (2017) Obesity-related parameters and colorectal adenoma development. J Gastroenterol. doi:10.1007/s00535-017-1319-0 10.1007/s00535-017-1319-0 28197803

[82] Laiyemo AO, Doubeni C, Badurdeen DS, Murphy G, Marcus PM, Schoen RE, Lanza E, Smoot DT, Cross AJ (2012). Obesity, weight change, and risk of adenoma recurrence: a prospective trial. Endoscopy.

[83] Taniguchi L, Higurashi T, Uchiyama T, Kondo Y, Uchida E, Uchiyama S, Jono F, Hamanaka J, Kuriyama H, Hata Y (2014). Metabolic factors accelerate colorectal adenoma recurrence. BMC Gastroenterol.

[84] Kim MC, Jung SW, Kim CS, Chung TH, Yoo CI, Park NH (2012). Metabolic syndrome is associated with increased risk of recurrent colorectal adenomas in Korean men. Int J Obes.

[85] Kim S, Baron JA, Mott LA, Burke CA, Church TR, McKeown-Eyssen GE, Cole BF, Haile RW, Sandler RS (2006). Aspirin may be more effective in preventing colorectal adenomas in patients with higher BMI (United States). Cancer Causes Control.

[86] Comstock SS, Xu D, Hortos K, Kovan B, McCaskey S, Pathak DR, Fenton JI (2016). Association of serum cytokines with colorectal polyp number and type in adult males. Eur J Cancer Prev.

[87] Gucalp A, Iyengar NM, Hudis CA, Dannenberg AJ (2016). Targeting obesity-related adipose tissue dysfunction to prevent cancer development and progression. Semin Oncol.

[88] Sax AT, Jenkins DG, Devin JL, Hughes GI, Bolam KA, Skinner TL (2014). The insulin-like growth factor axis: a biological mechanism linking physical activity to colorectal cancer survival. Cancer Epidemiol.

[89] Flood A, Mai V, Pfeiffer R, Kahle L, Remaley AT, Lanza E, Schatzkin A (2007). Elevated serum concentrations of insulin and glucose increase risk of recurrent colorectal adenomas. Gastroenterology.

[90] Durai R, Yang W, Gupta S, Seifalian AM, Winslet MC (2005). The role of the insulin-like growth factor system in colorectal cancer: review of current knowledge. Int J Color Dis.

[91] Flood A, Rastogi T, Wirfalt E, Mitrou PN, Reedy J, Subar AF, Kipnis V, Mouw T, Hollenbeck AR, Leitzmann M (2008). Dietary patterns as identified by factor analysis and colorectal cancer among middle-aged Americans. Am J Clin Nutr.

[92] Jacobs ET, Martinez ME, Alberts DS, Ashbeck EL, Gapstur SM, Lance P, Thompson PA (2008). Plasma insulin-like growth factor I is inversely associated with colorectal adenoma recurrence: a novel hypothesis. Cancer Epidemiol Biomark Prev.

[93] Pollak M (2008). Insulin and insulin-like growth factor signalling in neoplasia. Nat Rev Cancer.

[94] Wilhelmsen M, Kring T, Jorgensen LN, Madsen MR, Jess P, Bulut O, Nielsen KT, Andersen CL, Nielsen HJ (2014). Determinants of recurrence after intended curative resection for colorectal cancer. Scand J Gastroenterol.

[95] Li Y, Lyu Z, Zhao L, Cheng H, Zhu D, Gao Y, Shang X, Shi H (2015). Prognostic value of MGMT methylation in colorectal cancer: a meta-analysis and literature review. Tumour Biol.

[96] Meyerhardt JA, Catalano PJ, Haller DG, Mayer RJ, Macdonald JS, Benson AB, Fuchs CS (2003). Impact of diabetes mellitus on outcomes in patients with colon cancer. J Clin Oncol.

[97] Jeon JY, Jeong DH, Park MG, Lee JW, Chu SH, Park JH, Lee MK, Sato K, Ligibel JA, Meyerhardt JA (2013). Impact of diabetes on oncologic outcome of colorectal cancer patients: colon vs. rectal cancer. PLoS One.

[98] Siegel EM, Ulrich CM, Poole EM, Holmes RS, Jacobsen PB, Shibata D (2010). The effects of obesity and obesity-related conditions on colorectal cancer prognosis. Cancer Control.

[99] Wolpin BM, Meyerhardt JA, Chan AT, Ng K, Chan JA, Wu K, Pollak MN, Giovannucci EL, Fuchs CS (2009). Insulin, the insulin-like growth factor axis, and mortality in patients with nonmetastatic colorectal cancer. J Clin Oncol.

[100] Haydon AM, Macinnis RJ, English DR, Morris H, Giles GG (2006). Physical activity, insulin-like growth factor 1, insulin-like growth factor binding protein 3, and survival from colorectal cancer. Gut.

[101] Hanahan D, Weinberg RA (2011). Hallmarks of cancer: the next generation. Cell.

[102] Alonso S, Pascual M, Salvans S, Mayol X, Mojal S, Gil MJ, Grande L, Pera M (2015). Postoperative intra-abdominal infection and colorectal cancer recurrence: a prospective matched cohort study of inflammatory and angiogenic responses as mechanisms involved in this association. Eur J Surg Oncol.

[103] Demarzo MM, Martins LV, Fernandes CR, Herrero FA, Perez SE, Turatti A, Garcia SB (2008). Exercise reduces inflammation and cell proliferation in rat colon carcinogenesis. Med Sci Sports Exerc.

[104] Pedersen BK (2011). Exercise-induced myokines and their role in chronic diseases. Brain Behav Immun.

[105] Aoi W, Naito Y, Takagi T, Tanimura Y, Takanami Y, Kawai Y, Sakuma K, Hang LP, Mizushima K, Hirai Y (2013). A novel myokine, secreted protein acidic and rich in cysteine (SPARC), suppresses colon tumorigenesis via regular exercise. Gut.

[106] Songsorn P, Ruffino J, Vollaard NB (2017). No effect of acute and chronic supramaximal exercise on circulating levels of the myokine SPARC. Eur J Sport Sci.

[107] Grimm M, Lazariotou M, Kircher S, Hofelmayr A, Germer CT, von Rahden BH, Waaga-Gasser AM, Gasser M (2011). Tumor necrosis factor-alpha is associated with positive lymph node status in patients with recurrence of colorectal cancer-indications for anti-TNF-alpha agents in cancer treatment. Cell Oncol (Dordr).

[108] Aoi W, Naito Y, Takagi T, Kokura S, Mizushima K, Takanami Y, Kawai Y, Tanimura Y, Hung LP, Koyama R (2010). Regular exercise reduces colon tumorigenesis associated with suppression of iNOS. Biochem Biophys Res Commun.

[109] Ziech D, Franco R, Pappa A, Panayiotidis MI (2011). Reactive oxygen species (ROS)—induced genetic and epigenetic alterations in human carcinogenesis. Mutat Res.

[110] Radak Z, Chung HY, Goto S (2008). Systemic adaptation to oxidative challenge induced by regular exercise. Free Radic Biol Med.

[111] Barrasa JI, Olmo N, Lizarbe MA, Turnay J (2013). Bile acids in the colon, from healthy to cytotoxic molecules. Toxicol in Vitro.

[112] Wertheim BC, Martinez ME, Ashbeck EL, Roe DJ, Jacobs ET, Alberts DS, Thompson PA (2009). Physical activity as a determinant of fecal bile acid levels. Cancer Epidemiol Biomark Prev.

[113] Duggan C, Tapsoba JD, Wang CY, Campbell KL, Foster-Schubert K, Gross MD, McTiernan A (2016). Dietary weight loss, exercise, and oxidative stress in postmenopausal women: a randomized controlled trial. Cancer Prev Res (Phila).

[114] Morikawa T, Kuchiba A, Qian ZR, Mino-Kenudson M, Hornick JL, Yamauchi M, Imamura Y, Liao X, Nishihara R, Meyerhardt JA (2012). Prognostic significance and molecular associations of tumor growth pattern in colorectal cancer. Ann Surg Oncol.

[115] Jia M, Gao X, Zhang Y, Hoffmeister M, Brenner H (2016). Different definitions of CpG island methylator phenotype and outcomes of colorectal cancer: a systematic review. Clin Epigenetics.

[116] Toyota M, Ahuja N, Ohe-Toyota M, Herman JG, Baylin SB, Issa JP (1999). CpG island methylator phenotype in colorectal cancer. Proc Natl Acad Sci U S A.

[117] Slattery ML, Herrick JS, Pellatt DF, Stevens JR, Mullany LE, Wolff E, Hoffman MD, Samowitz WS, Wolff RK (2016). MicroRNA profiles in colorectal carcinomas, adenomas and normal colonic mucosa: variations in miRNA expression and disease progression. Carcinogenesis.

[118] Slattery ML, Herrick JS, Mullany LE, Stevens JR, Wolff RK (2017). Diet and lifestyle factors associated with miRNA expression in colorectal tissue. Pharmgenomics Pers Med.

[119] Pritchard CC, Cheng HH, Tewari M (2012). MicroRNA profiling: approaches and considerations. Nat Rev Genet.

[120] Koshiol J, Wang E, Zhao Y, Marincola F, Landi MT (2010). Strengths and limitations of laboratory procedures for microRNA detection. Cancer Epidemiol Biomark Prev : Publ Am Assoc Cancer Res Cosponsored Am Soc Prev Oncol.

[121] Chugh P, Dittmer DP (2012). Potential pitfalls in microRNA profiling. Wiley Interdiscip Rev RNA.

[122] Courneya KS, Vallance JK, Culos-Reed SN, McNeely ML, Bell GJ, Mackey JR, Yasui Y, Yuan Y, Matthews CE, Lau DC (2012). The Alberta Moving Beyond Breast Cancer (AMBER) cohort study: a prospective study of physical activity and health-related fitness in breast cancer survivors. BMC Cancer.

